# Supplementary material for: Plasmon-enhanced stimulated Raman scattering microscopy with single-molecule detection sensitivity
Source: Nat Commun. 2019 Nov 21;10:5318. doi: 10.1038/s41467-019-13230-1 (PMC6872561; doi:10.1038/s41467-019-13230-1)
Supplement: Supplementary file 1 — Supplementary Information [file 41467_2019_13230_MOESM1_ESM.pdf]

Supplementary Information

Plasmon-enhanced Stimulated Raman Scattering Microscopy with Single-molecule Detection Sensitivity

Cheng Zong et al.

Supplementary Figure

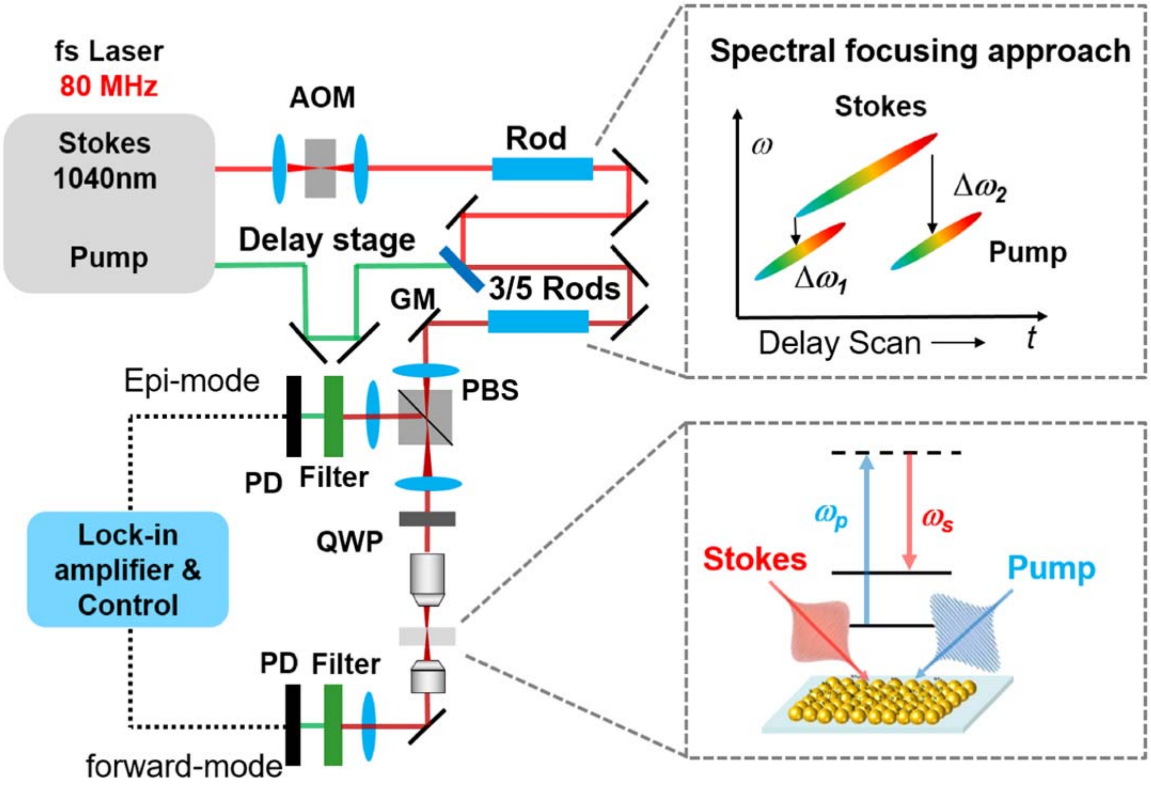

Supplementary Figure 1. The scheme of a hyperspectral plasmon-enhanced stimulated Raman scattering microscope. Laser system: 80 MHz tunable femtosecond laser. AOM: acousto-optic modulator. GM: 2D galvo mirror. PBS: polarizing beam splitter; QWP: quarter wave plate; PD: photodiode. For spectral focusing, three or five rods were used in combined path and one rod was used in Stokes path. In this way, the pump and Stokes pulses were chirped to achieve a constant instantaneous frequency difference that drives a single Raman coherence. A series of Raman shifts were generated by scanning the delay stage.

Supplementary Figure

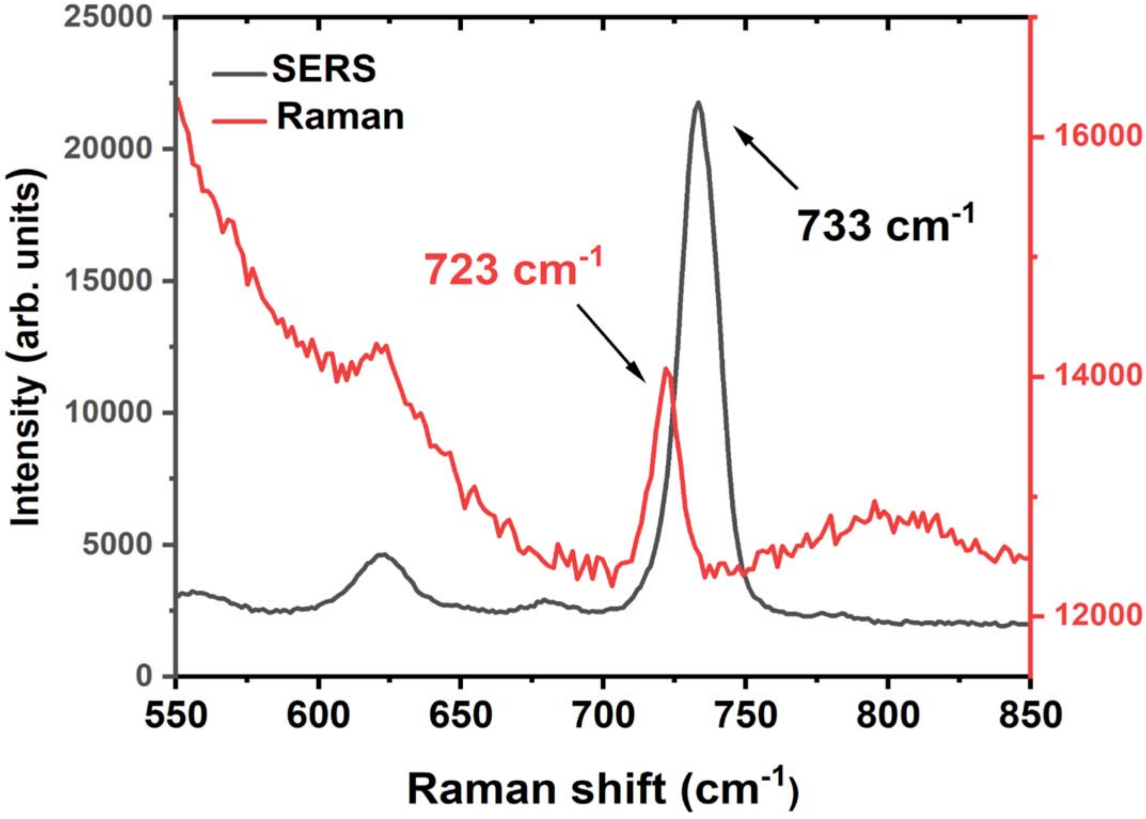

Supplementary Figure 2. The SERS spectrum of adenine adsorbed on Au NPs aggregation substrate and the Raman spectrum of adenine solution. The SERS spectrum (black) of adenine adsorbed on Au NPs aggregation substrate (5 mM in solution) has a peak at 733 cm^{-1}. The Raman spectrum (red) of 5 mM adenine solution has a peak at 723 cm^{-1}. The SERS spectrum was recorded with 5 s integration time, with a 50× objective and a 0.5 mW laser power at 785 nm. The Raman spectrum was recorded with 30 s integration time, with a 40× objective and an 80 mW laser power at 532 nm. This 10 cm^{-1} blue shift is due to the formation of metal-adenine complex.[1].

Supplementary Figure

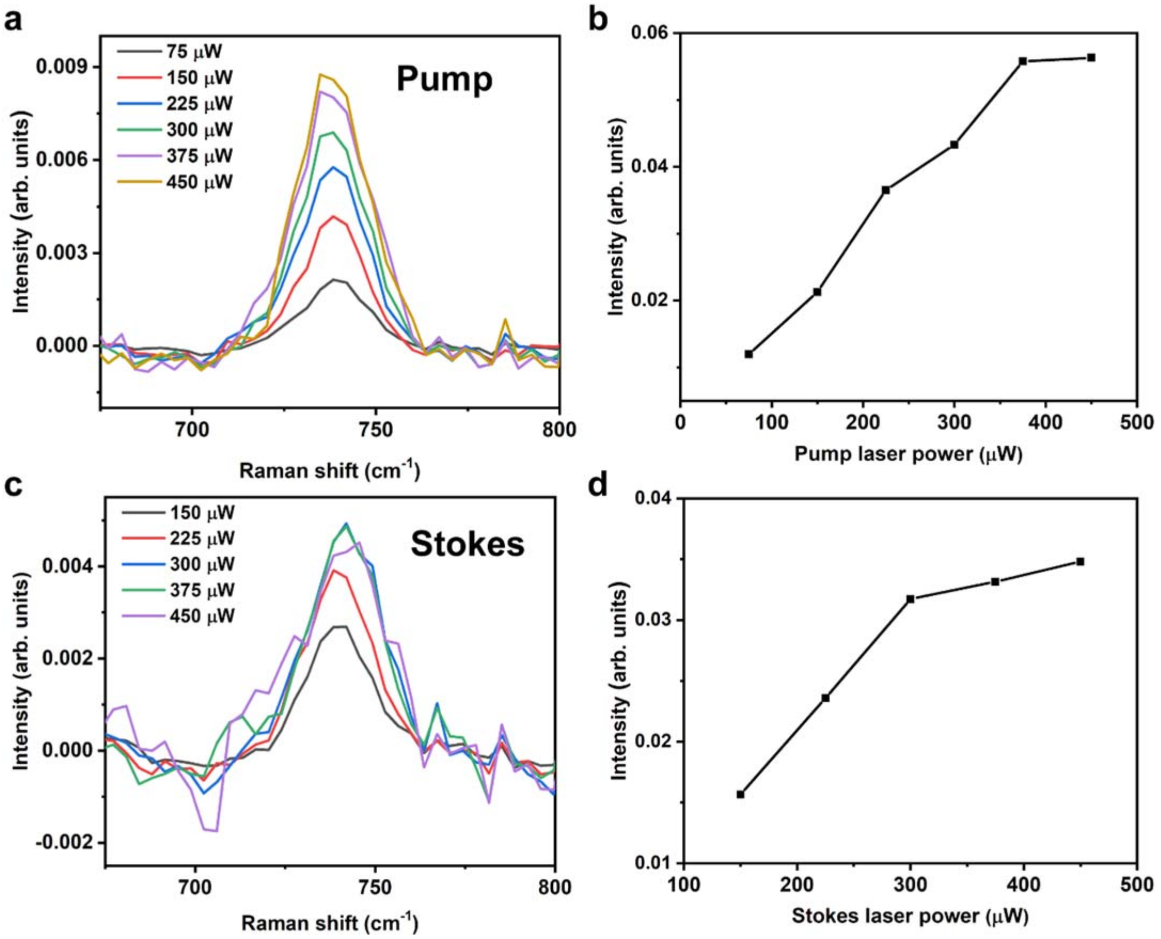

Supplementary Figure 3. **Dependence of plasmon-enhanced stimulated Raman scattering (PESRS) signal on pump and Stokes laser power.** (a) The pump power dependent PESRS spectra of adenine adsorbed on Au NPs aggregation substrate at the same position. The Stokes power was kept at 150 μW. (b) The peak area at 733 cm^{-1} of adenine obtained from (a) vs. pump power. (c) The Stokes power dependent PESRS spectra of adenine adsorbed on Au NPs aggregation substrate at the same position. The pump power was kept at 150 μW. (d) The peak area at 733 cm^{-1} of adenine obtained from (c) vs. Stokes power. The power value was the power at the sample. The PESRS signal has a linear relationship with the pump and Stokes power under a saturation power threshold (the sum of two laser power is c.a. 600 to 650 μW). The spectral features are similar. All laser powers, used in whole PESRS experiments, were kept below this threshold to minimize the photodamage of samples.

Supplementary Figure

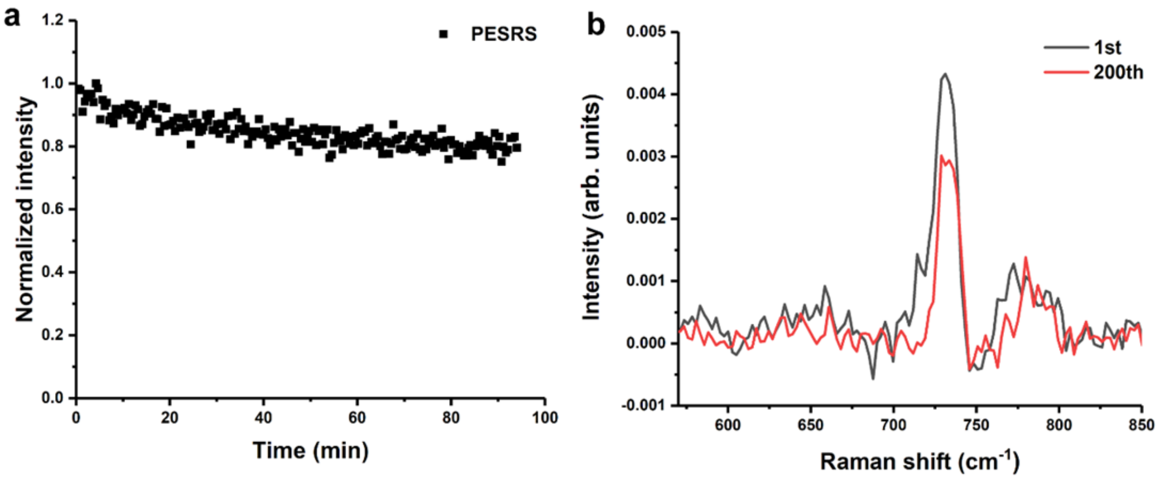

Supplementary Figure 4. The photostability of PESRS signal in a 1.5 h continual exposure. (a) The time-series of PESRS signal of adenine in 1.5 hour at the same location. About 20% of PESRS signal is lost in 1.5 h. (b) The 1st and 200th PESRS spectrum of adenine. 10 µL of 1 mM adenine was dropped into centrifuged Au NPs and was dry in vacuum. The power of pump and Stokes was 0.15 mW. Image area: 30 µm × 30 µm with 300 nm step size. Pixel dwell time: 10 µs. It took 0.47 min for one hyperspectral data. 200 hyperspectral data were continually obtained in 94 min. Time-dependent hyperspectral data cubes were denoised via BM4D and subtracted background.

Supplementary Figure

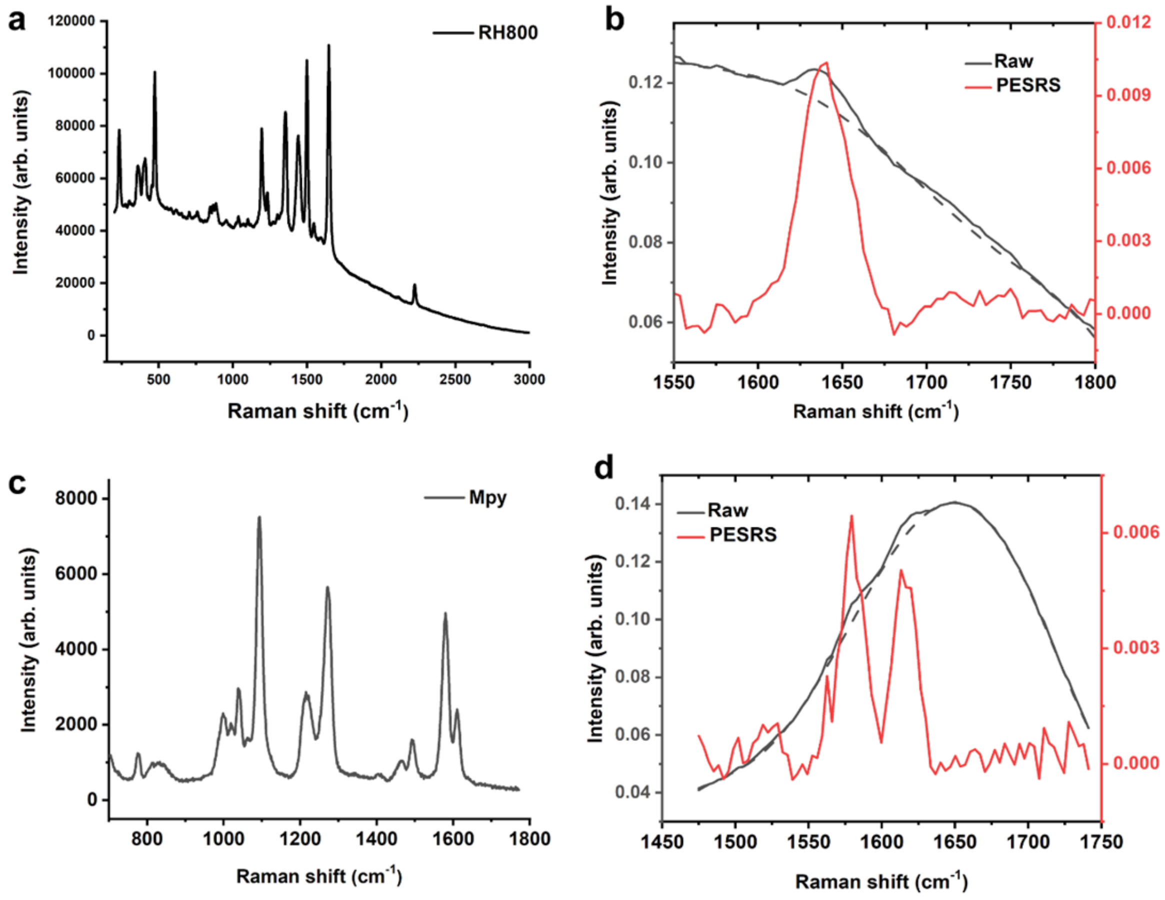

Supplementary Figure 5. SERS and PESRS spectra of Rhodamine 800 (RH800) and 4-mercaptopyridine (Mpy). (a) The SERS spectrum of RH800 adsorbed on Au NPs. The spectrum was recorded with 10 s integration time with a 50× objective and a 3 µW laser power at 785 nm. (b) Original PESRS spectrum (black solid) and fitted background (black dash) of RH800 adsorbed on Au NPs. The background-subtracted PESRS spectrum (red) of RH800. 75 µW pump laser (888 nm) and 50 µW Stokes laser were used. 10 µL of 85 µM RH800 was dropped into centrifuged Au NPs sol and was dry in vacuum. (c) The SERS spectrum of Mpy adsorbed on Au NPs. The spectrum was recorded with 2 s integration time with a 50× objective and a 300 µW laser power at 785 nm. (d) Original PESRS spectrum (black solid) and fitted background (black dash) of Mpy adsorbed on Au NPs. The background-subtracted PESRS spectrum (red) of Mpy. 150 µW pump laser (891 nm) and 150 µW Stokes laser were used. 10 µL of 5.7 mM Mpy was dropped into centrifuged Au NPs sol and was dry in vacuum. This result indicated that our method can obtain PESRS spectra from a variety of molecules.

Supplementary Figure

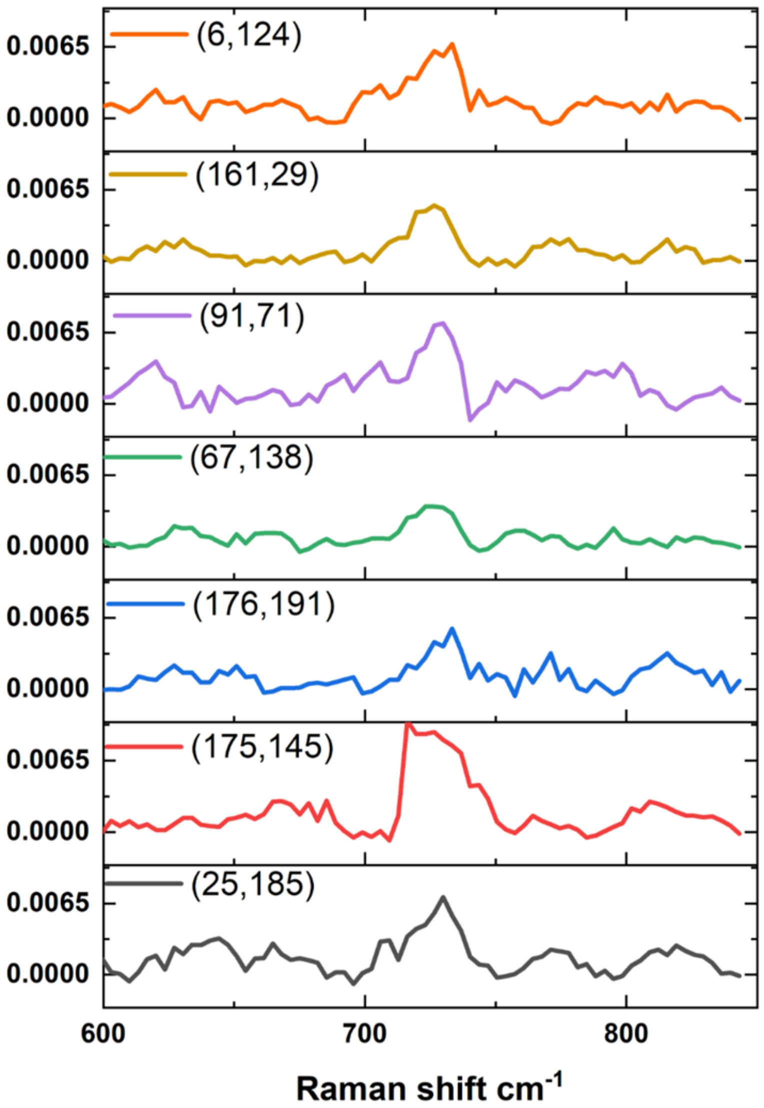

Supplementary Figure 6. Representative single-pixel PESRS spectra of adenine from a single image indicate good reproducibility. Representative single-pixel PESRS spectra of adenine, obtained from aggregated Au NPs substrate (the same hyperspectral data cube in Figure 2). The labels of each spectrum indicate the *X*-*Y* pixel coordinate.

Supplementary Note 1. The estimation of local enhancement factor of PESRS.

In stimulated Raman scattering, the signal intensity is calculated as[2]:

$$I = N \times \sigma \times P \times S$$

Where, I is the intensity, N is the number of molecules under the laser spot, σ is the molecular Raman scattering cross-section. P is the pump laser power. S is the Stokes laser power. Enhancement factor (EF) of PESRS relative to normal SRS is defined as a ratio of PESRS over SRS cross-sections ($EF = \sigma_{PESRS}/\sigma_{SRS}$). To estimate the EF of PESRS, we calculated the power- and concentration- averaged intensity between PESRS and SRS as following:

$$\sigma_{PESRS} = \frac{I_{PESRS}}{N_{PESRS} \times P_{PESRS} \times S_{PESRS}}$$

$$\sigma_{SRS} = \frac{I_{SRS}}{N_{SRS} \times P_{SRS} \times S_{SRS}}$$

Thus ,

$$EF = \frac{\sigma_{PESRS}}{\sigma_{SRS}} = \frac{I_{PESRS}}{I_{SRS}} \times \frac{N_{SRS}}{N_{PESRS}} \times \frac{P_{SRS}}{P_{PESRS}} \times \frac{S_{SRS}}{S_{PESRS}}$$

As shown in **Supplementary Figure 7**, the SRS spectrum (average 200 × 200 pixel area spectra) of 5 mM adenine solution was measured at the power of 15 mW (Pump) and 100 mW (Stokes) and the PESRS spectrum (average 3×3 pixel area spectra) of adsorbed adenine was measured at the power of 0.5 mW (Pump) and 0.5 mW (Stokes). We assumed the size of the laser spot was 500 nm, the size of adenine was 0.5 nm^2 per molecule. Au NPs was a monolayer under the laser spot and a monolayer adenine adsorbed on Au NPs surface. The number of molecules in detection volume (c.a. 30 μm × 30 μm × 1 μm = 900 $μm^3$) was about 2.7×10^9 for SRS detection. The number of molecules on the surface can be estimated as about 1.6×10^6 for PESRS. In this way, the local enhancement factor of PESRS was about 7×10^7.

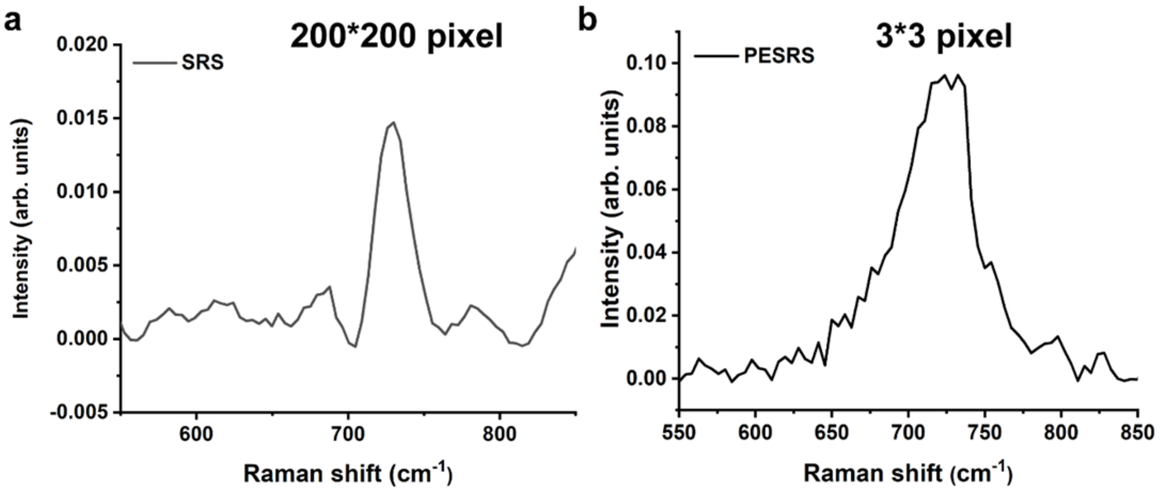

Supplementary Figure 7. **PESRS and SRS spectra of adenine.** (a) The SRS spectrum of 5 mM adenine solution (average 200×200 pixel area spectra). Pump power: 15mW, Stokes power: 100 mW. (b) The PESRS spectrum of adsorbed adenine on Au NPs-SiO_2 substrate (average 3×3 pixel area spectra). Pump power: 0.5 mW, Stokes power: 0.5 mW.

Supplementary Figure

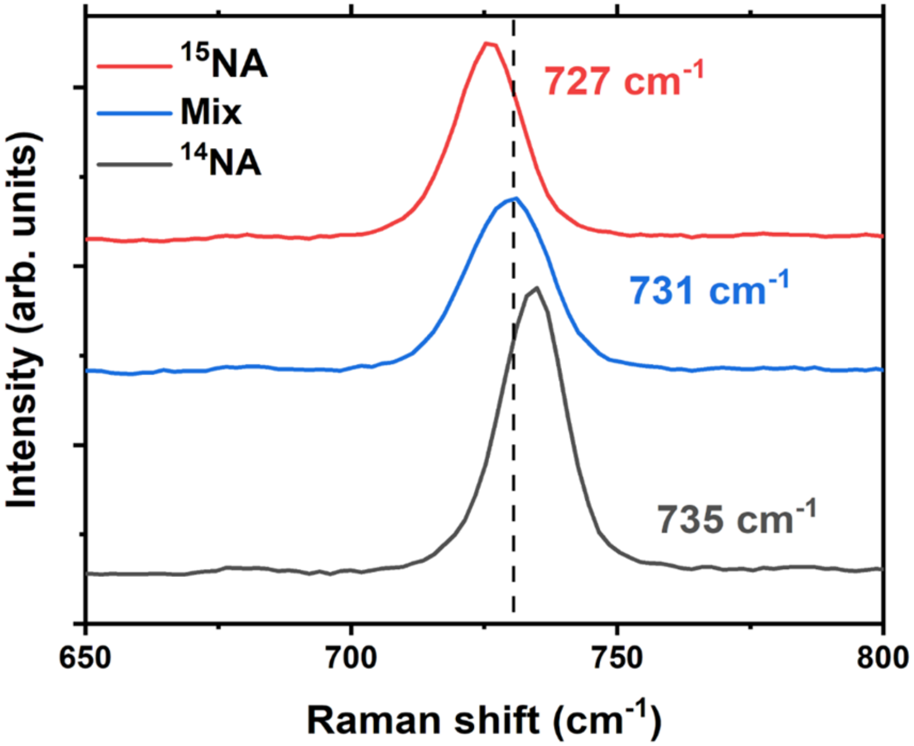

Supplementary Figure 8. **The SERS spectra of pure ^{14}NA, pure ^{15}NA and their equimolar mixture adsorbed on Au NPs aggregation substrates.** The SERS spectra of pure ^{14}NA, pure ^{15}NA and their equimolar mixture adsorbed on Au NPs aggregation substrates (1 mM in solution). The pure ^{14}NA SERS spectrum has a peak at 735 cm^{-1}, the pure ^{15}NA SERS spectrum has a peak at 727 cm^{-1}, and the equimolar mixture SERS spectrum has a peak at 731 cm^{-1}. These single isotope SERS spectra match well with the corresponding PESRS spectra.

Supplementary Figure

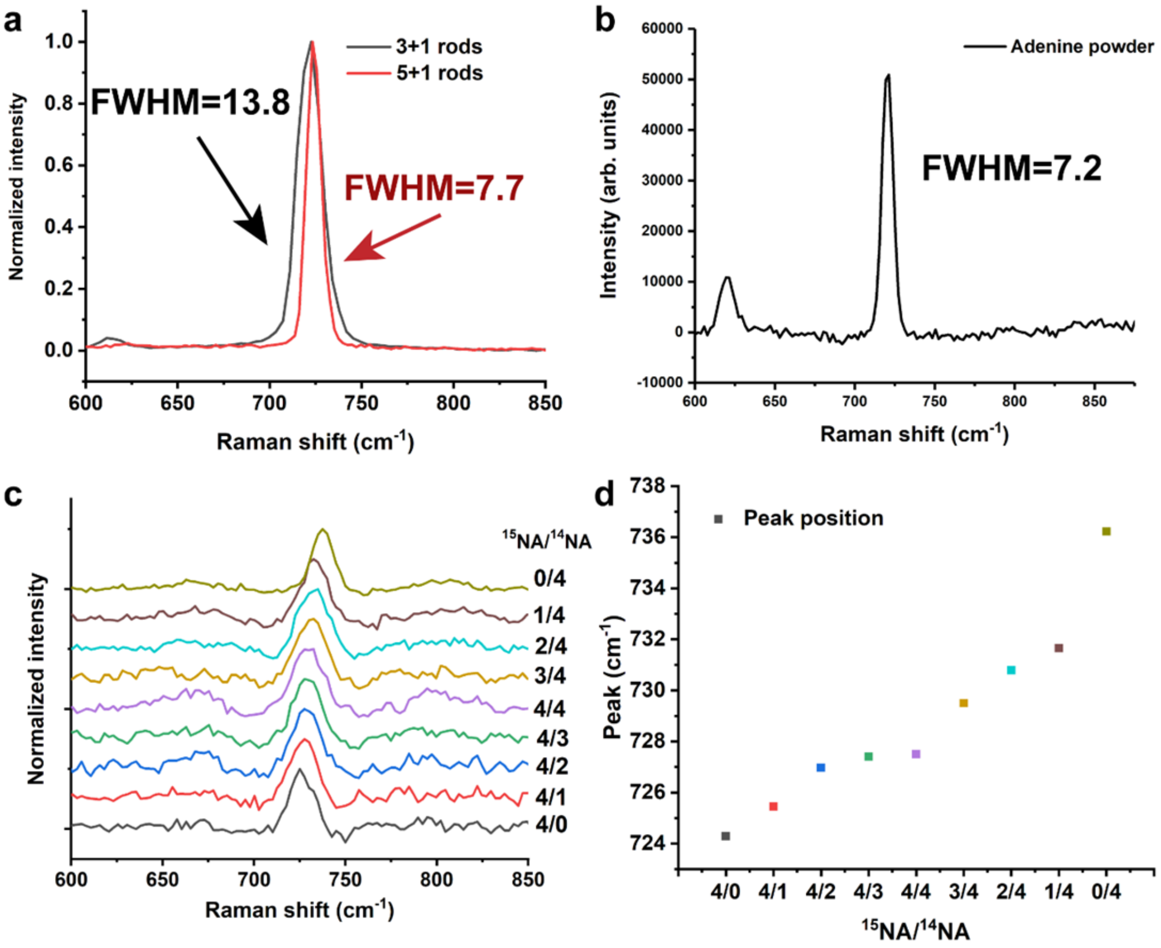

Supplementary Figure 9. Spectral resolution of spectral focusing SRS microscopy. (a) The SRS spectra and corresponding FWHM of adenine powder measured by different chirped laser. 3+1 rods: 3 rods in combined light path and 1 rod in Stokes light path. 5+1 rods: 5 rods in combined light path and 1 rod in Stokes light path. (b) The spontaneous Raman spectrum of adenine powder measured by Renishaw inVia Raman microscope with 1200/mm grating. Laser: 633 nm. (c) PESRS spectra as a function of concentration ratio of ^{15}NA and ^{14}NA. (d) PESRS peak positions of mixture samples as a function of concentration ratio of ^{15}NA and ^{14}NA. With the increasing of ^{14}NA, the PESRS peaks of mixture shift to high wavenumber. The PESRS spectra were measured by 5+1 rods. This result indicates that with a spectral resolution of 7 cm^{-1}, our PESRS microscope is able to distinguish the concentration ratio of ^{15}NA and ^{14}NA.

Supplementary Figure

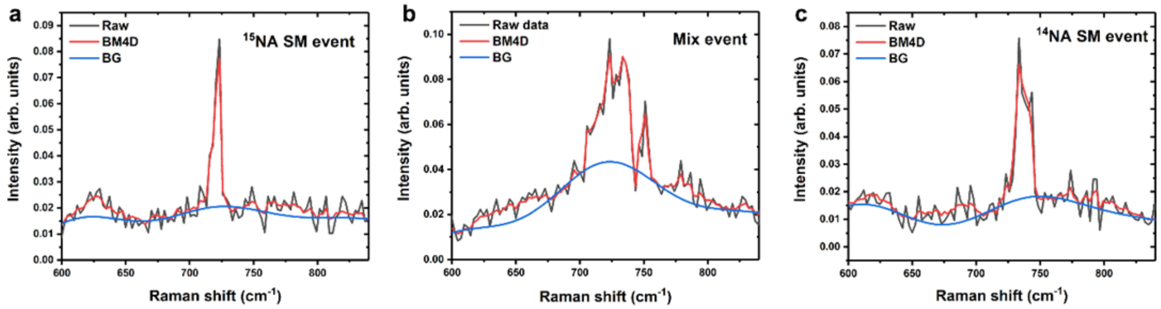

Supplementary Figure 10. The corresponding single molecule spectra with and without denoising and fitting background. The corresponding single-pixel PESRS spectra of ^{15}NA single molecule event (a), mix event (b), and ^{14}NA single molecule event (c) without denoising, after denoising and fitting background in Figure 4b.

Supplementary Figure

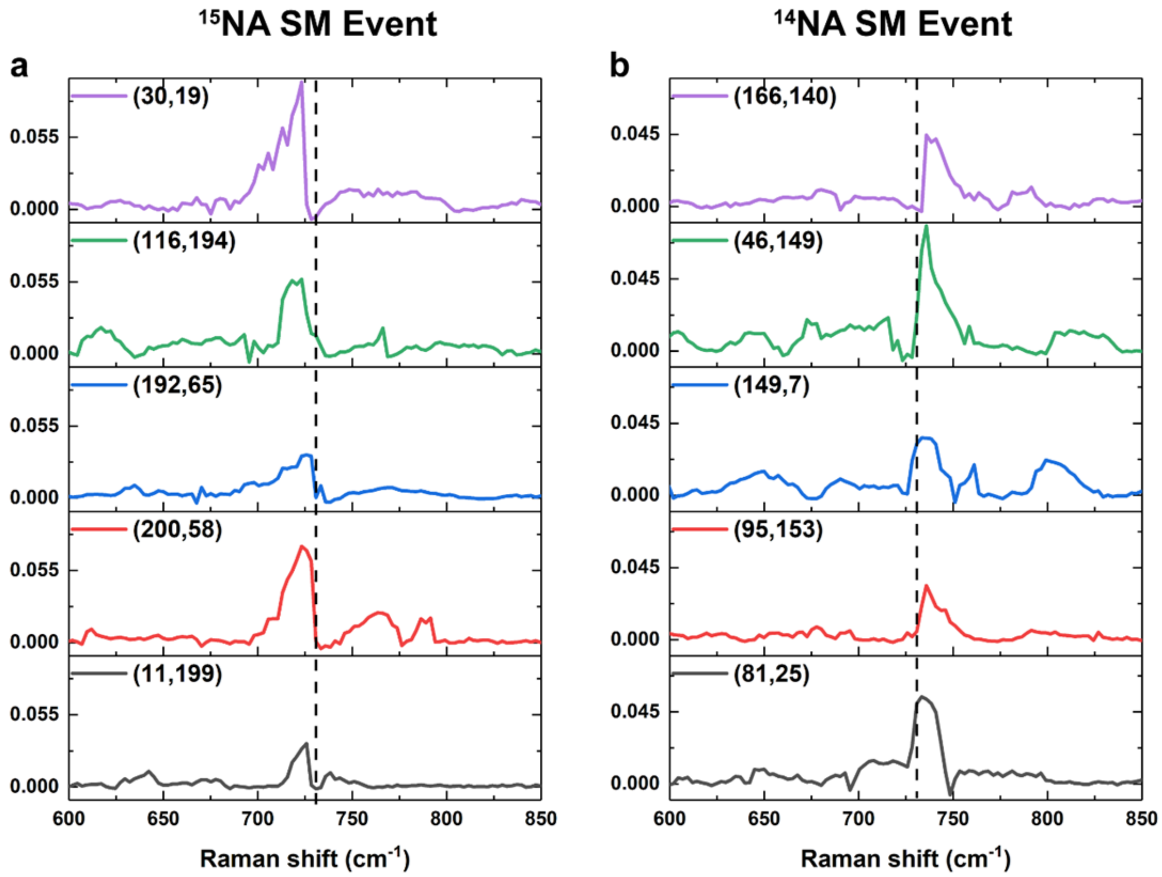

Supplementary Figure 11. The representative single-molecule events of adenine in a PESRS image. The representative single-molecule (SM) events from 50 nM adenine sample. Left column: pure ^{15}NA single-molecule events, Right column: pure ^{14}NA single-molecule events. The vertical dash lines indicate the position of 730 cm^{-1}. Legends indicate the *X*-*Y* coordinate in PESRS image.

Supplementary Note 2. The simulated single-molecule PESRS data

Here, we introduced a model example to describe the statistics of single-molecule PESRS signal in a hot spot. This model was based on the previous bianalyte approach mode developed by Eric Le Ru, PG Etchegoin, et at.[3] First, we used the boundary element method approach[4-6] to calculate a local electric field distribution on a representative hot spot. Supplementary Figure 12a presented the simulated local electric field distribution of the representative hot spot (a dimer formed by two 60 Au NPs with a 1 nm gap). Then, we assumed that we had a certain number of molecules of two isotopic adenines (N_{14} and N_{15}) on the hot spot. We generated random locations of molecules in the hot spot and every molecule felt a corresponding local electric field in the hot spot.[7] Then, we calculated the total intensity produced by each type of molecules (I_{14} and I_{15}) by summing over the corresponding intensity of every molecules. Because the Raman cross section of ^{14}NA and ^{15}NA were the same, the ratio of $I_{14}/(I_{14}+I_{15})$ was also the ratio of the average number of ^{14}NA contributing to the signal. We repeated this process for many times (as a large number of events). In order to mimic experiment condition, we only counted the events above a threshold (0.2 % of maximum total intensity) and obtained the histogram for relative contribution of ^{14}NA. In addition, we analyzed the number of signal-dominated molecules, which contribute 80 % of total signal, in every single molecule events (ratio ≈ 0 and ≈ 1). **Supplementary Figure 12** shows the the simulated results for repeating 100000 events with $N_{14}=N_{15}=10$ (b), 80 (c), and 1000 (d) molecules, respectively. For $N_{14}=N_{15}=10$, as shown in **Supplementary Figure 12b**, events were dominant by the ratio ≈ 0 and ≈ 1. **Supplementary Figure 12e** indicates that 90 % single molecule events (the ratio ≈ 0 and ≈ 1) was mainly contributed by 1 molecule for 10 molecules simulation. As shown in **Supplementary Figure 12c**, for $N_{14}=N_{15}=80$, inevitably, there were more mixed-signal events than that in $N_{14}=N_{15}=10$. However, **Supplementary Figure 12f** clearly indicates that 55 % single molecules events were mainly contributed by 1 molecule. This result indicated that the single molecule events in 80 molecules simulation can be attributed to real single-molecule events with a high probability. The many-molecules regime, $N_{14}=N_{15}=1000$. The

histogram (**Supplementary Figure 12d**) looks like a Gaussian distribution centered at the ratio=0.5. Most of events have contributions from many molecules. As shown in **Supplementary Figure 12c**, our 50 nM experimental result (green) matched well the simulated result (purple) of 80 molecules. This result indicated that our experiment has achieved a single-molecules detection regime.

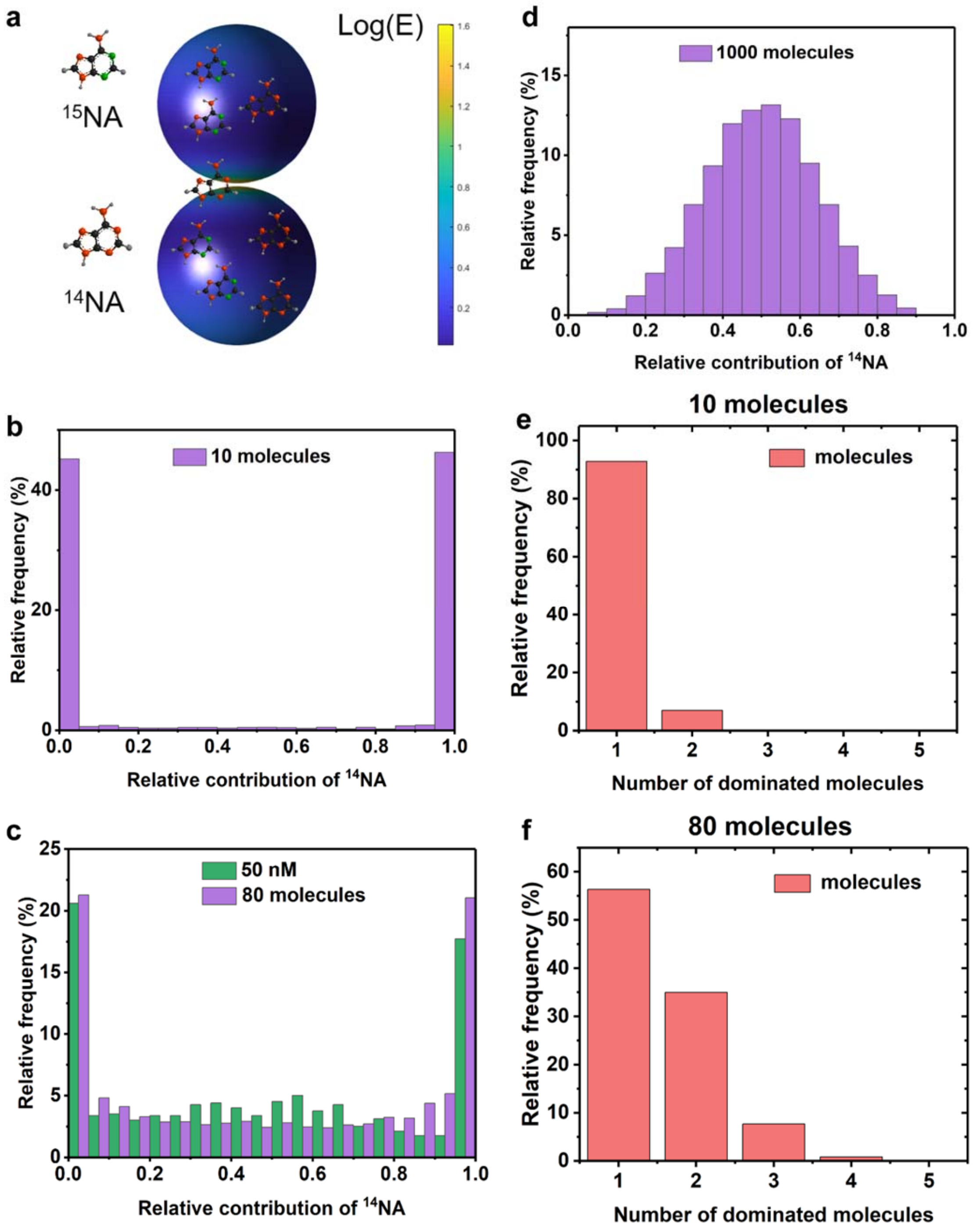

Supplementary Figure 12. The simulated single-molecule PESRS data (a) The local electric field distribution of the hot spot. The hot spot was formed by two 60 nm Au NPs with a 1 nm gap. Each molecule adsorbed on a certain location can experience a corresponding local electric field. (b & d) The histograms (purple) of the relative contribution of ^{14}NA in the simulated results with N_{14}=N_{15}=10 (b), 1000 (d) molecules, respectively. (c) The histograms of the relative contribution of ^{14}NA in the 50 nM mixture sample (green) and simulated result (purple) of N_{14}=N_{15}=80. (e-f) The histogram of number of dominated molecules which contribute 80 % total signal in single molecule events with N_{14}=N_{15}=10 (e), 80 (f).

As a control experiment, we measured 50 nM pure ^{14}NA and pure ^{15}NA samples, respectively. **Supplementary Figure. 13a&b** shows the histograms of relative contribution of ^{14}NA of isotopic pure ^{14}NA and pure ^{15}NA samples. The histogram of pure ^{14}NA and pure ^{15}NA sample dominated by the ratio ≈ 1 (**Supplementary Figure 13b**) and ≈ 0 (**Supplementary Figure 13a**), respectively.

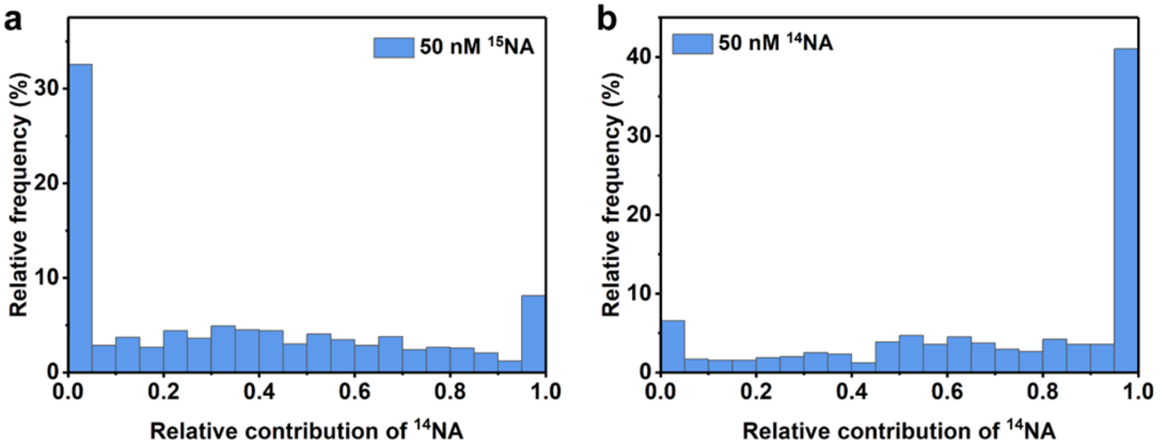

Supplementary Figure 13. The histograms of the relative contribution of ^{14}NA in pure sample. The histograms of the relative contribution of ^{14}NA in (a) the 50 nM ^{15}NA sample and (b) 50 nM ^{14}NA sample.

Supplementary Note 3. The reliability of multivariate curve resolution (MCR) analysis for Fano-shape spectra

To verify the reliability of multivariate curve resolution (MCR) analysis for Fano-shape spectra, we measured a 1 mM pure ^{14}NA, a pure ^{15}NA and their mixture sample. As we expected, the histogram of the relative contribution of ^{14}NA in 500 µM of mixture sample (**Supplementary Figure 14a**) looks like a Gaussian distribution center at the ratio=0.5. In addition, the histogram of 1 mM pure ^{14}NA sample was dominated by pure ^{14}NA signal (ratio ≈ 1), as show in **Supplementary Figure 14c**. While "mixture" signals also were observed in the histogram which might result from the various dispersive line shape of PESRS in different single-pixel spectra. Those various dispersive line shape depended on the local LSPR frequency and local enhancement. Similarly, in the pure ^{15}NA sample (**Supplementary Figure 14b**), a significant portion of signals was assigned to pure ^{15}NA (ratio ≈ 0) and little to ^{14}NA.

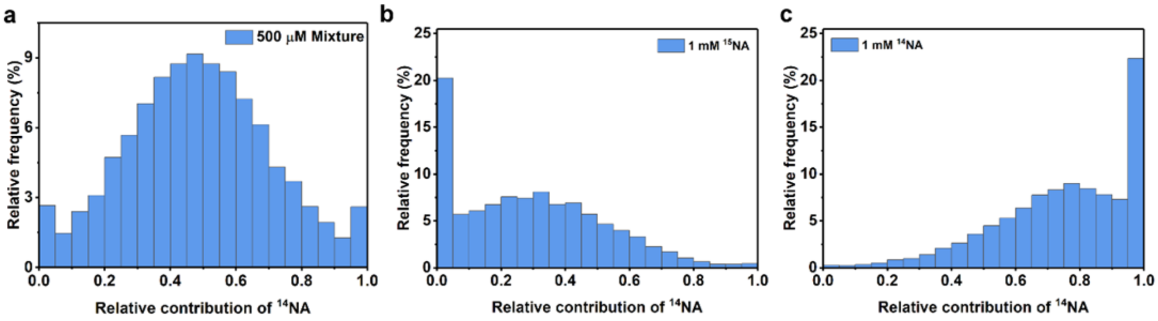

Supplementary Figure 14. The histograms of the relative contribution of ^{14}NA in ensemble pure and mixed sample. The histograms of the relative contribution of ^{14}NA in 500 µM the mixture of ^{14}NA and ^{15}NA sample (a), 1 mM ^{15}NA sample (b) and 1 mM ^{14}NA sample (c).

In addition, we used the Fano-line shape function ($\mathbf{f}(\boldsymbol{x}) = \boldsymbol{A}\left\{\frac{(\boldsymbol{q}+\frac{x-x0}{\Gamma/2})^2}{1+(\frac{x-x0}{\Gamma/2})^2}\right\}$) to fit the single pixel spectra of the 50 nM adenine sample. Here, $\boldsymbol{A}$ is the amplitude of each peak, $\boldsymbol{q}$ is the Fano asymmetry parameter, $\boldsymbol{x0}$ is the center frequency of vibrational feature, $\boldsymbol{x}$ is the Raman shift, $\boldsymbol{\Gamma}$ is the line width. We find that most of fitted Fano asymmetry parameter (q) are larger than 2 or smaller than -2 (**Supplementary Figure 15a**). Based on this result, we simulated a series of q

value dependent ^{14}NA and ^{15}NA spectra as shown in **Supplementary Figure 15b**. The simulation result show that when $q > 2$ or < -2, the different q value can induce the shift of peak position. While, ^{14}NA and ^{15}NA spectra still can be differentiated. Our results indicated that various dispersive line shapes of PESRS spectra slightly affect the Raman peak frequency. However, this slight frequency shift has no obvious impact on the molecular assignment between ^{14}NA and ^{15}NA.

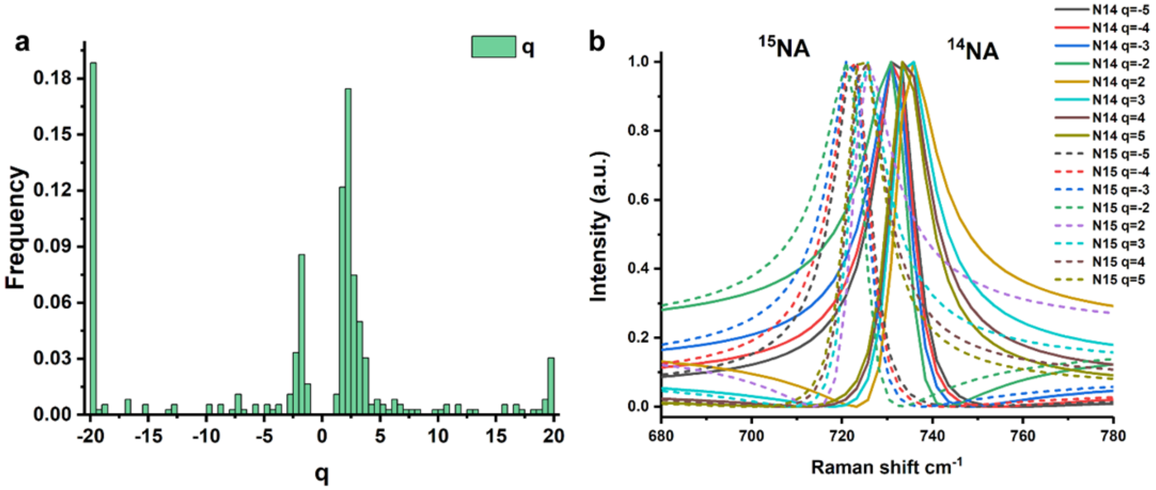

Supplementary Figure 15. Fano-line fitting and simulation spectra (a) The distribution of q value of PESRS spectra of 50 nM adenine. (b) Simulation ^{14}NA and ^{15}NA spectra with a function of Fano q parameter. Position: $x0$=733 cm^{-1} and 726 cm^{-1} for ^{14}NA and ^{15}NA, respectively. Width: Γ=10 cm^{-1}.

Supplementary Figure

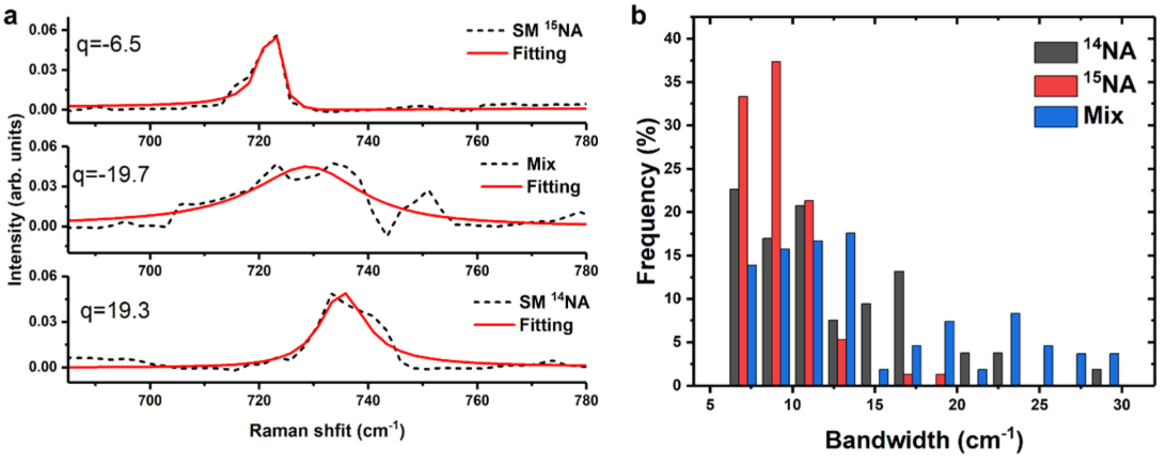

Supplementary Figure 16. Bandwidth statistics in a PESRS image. (a) The representative single-pixel PESRS spectra (dash lines) of ^{15}NA single molecule event, mix event, and ^{14}NA single molecule event fitted with a Fano-lineshape function (red lines). The fitting function was shown as following: $\mathbf{f}(\boldsymbol{x}) = \boldsymbol{A}\left\{\frac{(\boldsymbol{q}+\frac{x-x0}{\Gamma/2})^{2}}{1+(\frac{x-x0}{\Gamma/2})^{2}}\right\}$. Here, $\boldsymbol{A}$ is the amplitude of each peak, $\boldsymbol{q}$ is the Fano asymmetry parameter, $\boldsymbol{x0}$ is the center frequency of vibrational feature, $\boldsymbol{\Gamma}$ is the line width. Corresponding fitted $\boldsymbol{q}$ value indicated in text. (b) Histogram displaying the width of the peak bandwidth for SM ^{14}NA (black) events, SM ^{15}NA (red) events, and mix (blue) event. The bandwidth of mixed events (blue, average: 14.6±6.5 cm^{-1}) is larger than the bandwidths of single molecule spectra (black and red, average: 10.5±3.9 cm^{-1}).

Supplementary Note 4. The time-lapsed PESRS images collected on a 50 nM and 1 mM adenine sample

To further validate the single-molecules sensitivity of PESRS, we measured the time-series PESRS signal (as shown in **Supplementary Movie 2**) from a 50 nM adenine solution adsorbed on Au NPs at the same location. 10 µL of 50 nM adenine was dropped into centrifuged Au NPs and was dry in vacuum. The power of pump and Stokes was 0.15 mW. Image area: 30 µm × 30 µm with 300 nm step size. Pixel dwell time: 10 µs. It took 0.47 min for one hyperspectral data. 200 hyperspectral data were continually obtained in 94 min. Movie S2 shows the BM4D denoised and background-corrected PESRS signal. Previous time-series PESRS of 1 mM adenine (as shown in **Supplementary Movie 1**) was used as a control experiment.

As shown in Fig 4, representative time traces of 50 nM adenine (**Fig 4e**) show that strong Raman signal fluctuations during the PESRS measurements at the same locations. While, 1 mM adenine ensemble sample results (**Supplementary Figure 17**) show a stable intensity change. This spectral blinking phenomenon is consider an additional signature of the behavior of single molecule spectral sensitivity. In addition, **Fig 4e** shows representative photodamage processes of 50 nM adenine. The 50 nM sample exhibited a single-step photodamage process, while the ensemble molecule sample (1 mM) result shows a stable intensity trace (**Supplementary Figure 17**). Our time-series PESRS results exhibited characteristic single-molecules behaviors including blinking and single-step photodamage, which further verify the single-molecules sensitivity of PESRS.

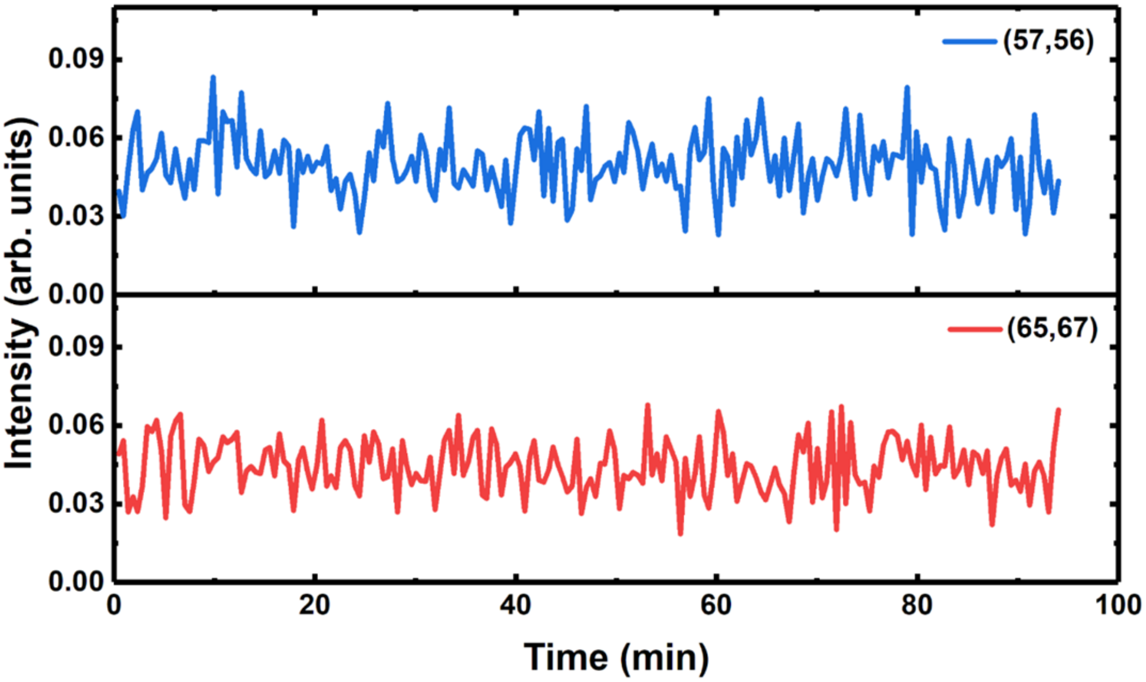

Supplementary Figure 17. The time-lapsed PESRS images. Representative time traces of PESRS spectra collected of 1 mM adenine solution showing relative stable intensity traces. The inside labels show the the *X*-*Y* coordinate where the spectra were recorded

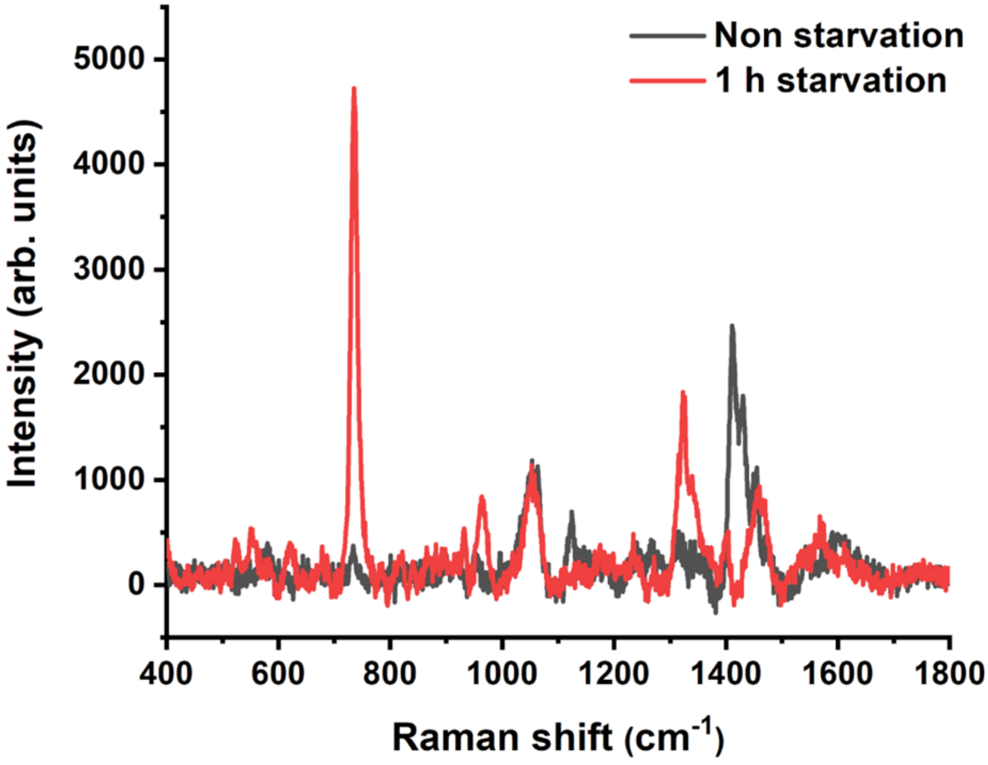

Supplementary Figure 18. SERS spectra of starved *S. aureus* and non-starved *S. aureus*. SERS spectra of starved *S. aureus* (red) and non-starved *S. aureus* (black). After 1 hour starvation in pure water, the SERS spectrum of *S. aureus* closely resembled the SERS of adenine. This result indicates that the adenine appeared at the outer layer of *S. aureus* and in the extracellular metabolome as resulting from the bacterial cell stress response to the no-nutrient, water-only environment. The spectra were recorded with 10 s integration time with a 20× objective and a 0.5 mW laser power at 785 nm.

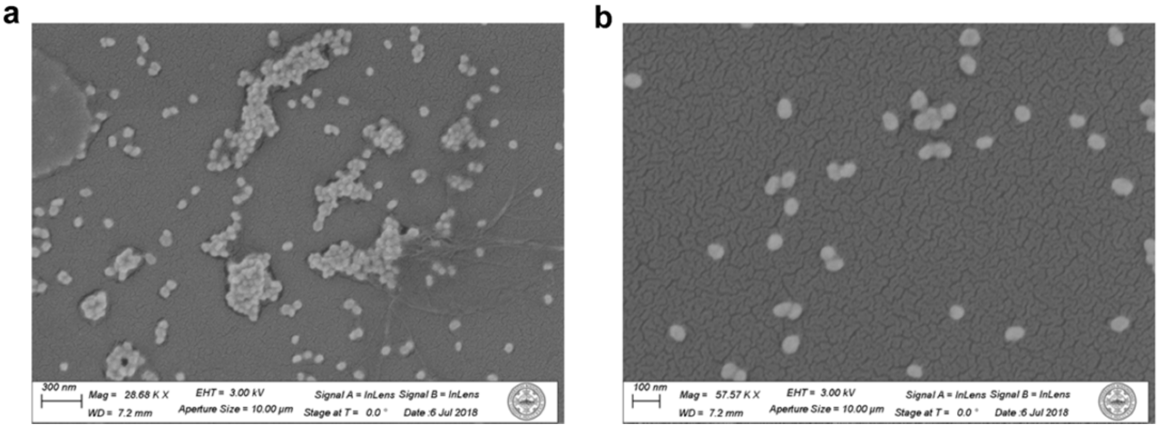

Supplementary Figure 19. SEM images of Au NPs colloid. The scale bar in (a) is 300 nm, and in (b) is 100 nm. The size of Au NPs is around 50 to 60 nm.

Supplementary References

1. Xiao, Y.-J., Chen, Y.-F. & Gao, X.-X. Comparative study of the surface enhanced near infrared Raman spectra of adenine and NAD+ on a gold electrode. *Spectrochim. Acta A* **55**, 1209-1218 (1999).
2. Cheng, J.-X. & Xie, X.S. Coherent Raman scattering microscopy. (CRC press, Boca Raton; 2016).
3. Etchegoin, P.G., Meyer, M., Blackie, E. & Le Ru, E.C. Statistics of single-molecule surface enhanced Raman scattering signals: Fluctuation analysis with multiple analyte techniques. *Anal. Chem.* **79**, 8411-8415 (2007).
4. De Abajo, F.G. & Howie, A. Retarded field calculation of electron energy loss in inhomogeneous dielectrics. *Phys. Rev. B* **65**, 115418 (2002).
5. De Abajo, F.G. Optical excitations in electron microscopy. *Rev. Mod. Phys.* **82**, 209 (2010).
6. Hohenester, U. & Trugler, A. Interaction of single molecules with metallic nanoparticles. *IEEE J. Sel. Top. Quantum Electron.* **14**, 1430-1440 (2008).
7. Prince, R.C., Frontiera, R.R. & Potma, E.O. Stimulated Raman scattering: from bulk to nano. *Chem. Rev.* **117**, 5070-5094 (2016).
