# Supplementary material for: Plasmon-enhanced stimulated Raman scattering microscopy with single-molecule detection sensitivity
Source: Nat Commun. 2019 Nov 21;10:5318. doi: 10.1038/s41467-019-13230-1 (PMC6872561; doi:10.1038/s41467-019-13230-1)
Supplement: Supplementary file 3 — Description of Additional Supplementary Files [file 41467_2019_13230_MOESM3_ESM.pdf]

Description of Additional Supplementary Files

File Name: Supplementary Movie 1

Description: Time-series PESRS image of 1 mM adenine adsorbed on surface. The color of each pixel represents the peak area at 733 cm-1 after BM4D denoising and background subtraction.

File Name: Supplementary Movie 2

Description: Time-series PESRS image of 50 nM adenine adsorbed on surface. The color of each pixel represents the peak area at 733 cm-1 after BM4D denoising and background subtraction.
