# Supplementary figures and images for: Plasmon-enhanced stimulated Raman scattering microscopy with single-molecule detection sensitivity
Source: Nat Commun. 2019 Nov 21;10:5318. doi: 10.1038/s41467-019-13230-1 (PMC6872561; doi:10.1038/s41467-019-13230-1)

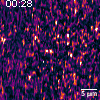

Supplement: Supplementary file 4 — Supplementary Movie 1 [file 41467_2019_13230_MOESM4_ESM.gif]

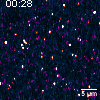

Supplement: Supplementary file 5 — Supplementary Movie 2 [file 41467_2019_13230_MOESM5_ESM.gif]
